# Complementary Feeding and Malnutrition among Infants and Young Children Aged 6–23 Months in Rural Areas of China

**DOI:** 10.3390/nu14091807

**Published:** 2022-04-26

**Authors:** Jing Feng, Zhaolong Gong, Yongjun Wang, Junsheng Huo, Qin Zhuo

**Affiliations:** 1 Key Laboratory of Trace Element Nutrition of National Health Commission, National Institute for Nutrition and Health, Chinese Center for Disease Control and Prevention, Beijing 100050, China; fengjing0921@163.com (J.F.); gongzl@ninh.chinacdc.cn (Z.G.); wangyongjun519@163.com (Y.W.); huojs@ninh.chinacdc.cn (J.H.); 2 Department of Clinical Nutrition, The First Affiliated Hospital of Shandong First Medical University & Shandong Provincial Qianfoshan Hospital, Jinan 250012, China

**Keywords:** malnutrition, early-life nutrition, micronutrient deficiencies, complementary feeding

## Abstract

This study investigated the nutrition and complementary feeding (CF) of infants and young children (IYC) aged 6–23 months in rural areas of China in 2018 and explored the relationship between CF and nutritional status. We measured the length and weight, calculated the z-scores, and detected micronutrients in the hair. The status of CF was obtained from the respondents by a 24-h dietary recall. IYC were classified into clusters using a two-step cluster analysis. The CF and nutritional status of each cluster were analyzed and compared. The prevalence of stunting, wasting, and overweight in the IYC in rural Chinese areas was 7.1%, 3.0%, and 3.7%, respectively. The median levels of Ca, Fe, and Zn in hair were 550.10 µg/g, 62.94 µg/g, and 132.86 µg/g, respectively. The prevalence of meeting the requirements of minimum dietary diversity (MDD), minimum meal frequency (MMF), and minimum acceptable diet (MAD) was 68.9%, 77.9%, and 46.4%, respectively. IYC with a higher prevalence of MDD, MMF, and MAD were more inclined to maintain a healthy status. The prevalence of undernutrition and overweight of 6- to 23-month-old IYC in rural areas of China was low. However, lack of trace elements was evident, and MAD prevalence remained low.

## 1. Introduction

Malnutrition refers to an insufficient, excessive, or unbalanced energy or nutrient intake. Malnutrition includes three categories: undernutrition or anthropometric failures [1] (including stunting, underweight, and wasting); micronutrient-related malnutrition, including micronutrient deficiencies (MNDs) and micronutrient excesses; and overweight or obesity [2], which occurs when the energy intake of food and beverages exceeds the energy needs of children. Being overweight or obese increases the risk of diet-related noncommunicable diseases later in life. Nutritional deficiencies in infancy also cause severe and irreversible physical and cognitive damage, which may last for life and affect the next generation [3,4]. Wasting and stunting are short- and long-term malnutrition indicators, respectively [4,5].

Stunting and wasting are major public health concerns. Globally, 52 million children under 5 years are wasted, 17 million are severely wasted, 155 million are stunted, and 41 million are overweight [2]. Some children even suffer from more than one form of malnutrition, such as stunting, overweight, and stunting accompanied by MNDs. During the past decades, the nutritional status of children under 5 in China has improved dramatically; however, gaps still existed between poor and non-poor areas. For example, in 2013, the prevalence of stunting, underweight, and wasting among children under 5 in poor areas were 18.7%, 5.2%, and 3.0%, respectively, in contrast to the prevalence of 8.1%, 2.4%, and 1.9% in China [6]. Malnutrition increases the incidence and severity of infections in children and delays recovery [7], and can even cause serious personal and social diseases and economic burdens [8,9,10]. The burden of malnutrition in children is mainly caused by poor dietary quality and intake. Nutrition-related factors account for 45% of deaths in children under 5 [2].

Nutritional status in the first 1000 days of life, from pregnancy to 2 years of age, affects the body’s metabolic model, which often lasts for a lifetime, influencing the risk of childhood and adult obesity and other diseases [11]. Nutritional factors in the first 1000 days of life include maternal nutrition during pregnancy, breastfeeding, formula feeding, and complementary feeding [12]. Breastfeeding and formula feeding are gradually reduced at 6 months, whereas the intake of solid or semi-solid foods increases [13]. Meanwhile, 6- to 23-month-old infants and young children (IYC) need more nutrition and energy for growth and development than any other time in their life. Nutrients in breast milk will no longer meet the rapid growth needs of IYC, so it is recommended to feed IYC complementary foods from 6 months. In Chinese rural areas, infants aged 6–23 months possess the highest prevalence of malnutrition among children under 5 years. Zhang, Y. et al. [9], through an investigation of 6570 children under 5 among 26 counties in poor rural areas of China in 2016, found that the prevalence of malnutrition in infants aged 6–11 months and 12–23 months was 17.4% and 27.9%, respectively. The prevalence of malnutrition in infants aged 6–23 months was significantly higher than that in infants aged 0–5 months and 24–60 months. WHO recommends that children start eating their first bite of solid food at 6 months old and continue breastfeeding until 2 years old to ensure that their nutritional intake provides sufficient energy for the developing brain and body development [14]. However, 44% of IYC aged 6–23 months had no access to fruits or vegetables globally, and 59% did not eat adequate eggs, dairy products, fish, or meat. Nearly two thirds of IYC aged 6–23 months did not receive the minimum diversified diet recommended for healthy growth and development [15]. What is worse, only one in five children aged 6–23 months from the poorest families and rural areas received the minimum diversified diet recommended for healthy growth and brain development [15]. A 50-year follow-up found that malnutrition during the critical period of brain development led to serious growth disorders, and maldevelopment in the first 1000 days of life had long-term adverse consequences for subsequent school education achievements and other human capital [16]. Intervention in the first two years of life is the best time to end the vicious cycle of malnutrition [17], and adequate feeding is important to ensure that nutritional needs are met. Ali, M. et al. even contended that underfeeding is the leading cause of premature death and malnutrition among young children in Pakistan [18]. More needs to be done to eliminate all forms of malnutrition by 2030 [19].

We hypothesized that the high prevalence of malnutrition in 6- to 23-month-old infants of Chinese rural areas was exceptionally relevant to the status of unqualified supplementary feeding. This study focused on the nutritional and complementary feeding status of IYC aged 6–23 months in rural areas of China in 2018. It explored the relationship between CF in the early stage of life and nutritional status in IYC, and discussed the influence on growth and development of IYC.

## 2. Materials and Methods

### 2.1. Study Design and Population

This study’s data were obtained from the Nutrition Improvement Project on Children in Poor Areas of China (NIPCPAC) in 2018 [20,21]. This study used multistage sampling, probability proportional to size (PPS) sampling, and random equidistant sampling to select samples. Five provinces of China named Guizhou, Henan, Xinjiang, Hubei, and Hebei were selected as the research provinces among the twenty-one provinces covered by NIPCPAC. One county was selected from each province. All towns were lined up by the net income per capita in the selected counties. One town was selected from each province using the PPS sampling method, in which the sampling probability was proportional to the number of live births in the town in 2018. Then, 3–5 sample villages per town were selected using the same PPS sampling method. Finally, all IYC aged 6–23 months in the sample villages in each town were ranked from youngest to oldest, and 60 IYC were sampled using a random equidistant sampling method. In addition, individuals with serious diseases that affect growth and development were excluded, such as congenital heart disease. Based on the sampling design and exclusion criteria above, 300 IYC aged 6–23 months were selected from each county. In addition, one third of the IYC were randomly selected from each sampled county for in-depth analysis, and laboratory testing content of biological samples, such as hair, was added. A total of 1527 IYC aged 6–23 months were included in the study; after data cleaning, there were 1522.

This project was reviewed and approved by the Ethics Committee of the Institute of Nutrition and Health of the Chinese Center for Disease Control and Prevention (No. 2018-017). All the caregivers provided written informed consent.

### 2.2. Data Collection

#### 2.2.1. Anthropometry

An intelligent physical examination instrument FSG-25-YE (BeiGao Corp., Shanghai, China) was used to measure the weight and length of the IYC. Body weight was measured and recorded to the nearest 0.05 kg; body length was recorded to the nearest 0.1 cm. The Ca, Fe, and Zn contents in the collected hair were measured using inductively coupled plasma mass spectrometry (ICP-MS; Huizhi Taikang Co., Ltd., Beijing, China).

#### 2.2.2. Questionnaires

Questionnaires designed by NIPCPAC experts were used to collect data on the essential characteristics of IYC, including age and sex. Parents’ and caregivers’ information, including their age, ethnicity, level of education, and occupation, was also obtained. The dietary status data were collected through a 24-h dietary recall of IYC respondents. The frequency of seven groups of complementary foods: grains, roots, and tubers; vitamin-A-rich fruits and vegetables (dark green, orange, and red fruits and vegetables, and red or orange tuber crops); other fruits and vegetables; fresh foods (meat, fish, poultry, and liver/organ meats); eggs; dairy products (milk, infant formula, yogurt, and cheese); and legumes and nuts were collected.

### 2.3. Grouping Criterion

A two-step cluster analysis was used to divide the data into clusters with elevated within-cluster homogeneity and between-cluster heterogeneity [22]. The IYC were categorized according to their z-scores or to Ca, Fe, and Zn levels in the hair. The differences in the participants’ clusters were subsequently compared.

### 2.4. Outcome Measures

We examined four child malnutrition outcome measures: stunting, underweight, wasting, and overweight. WHO Anthro was used to handle data and calculate the weight-for-length (WLZ), weight-for-age (WAZ), and length-for-age (LAZ) z-scores. Anthropometric failure outcomes were constructed based on the 2006 World Health Organization child growth standards [23]. Stunting was defined as an LAZ < −2 standard deviations (SDs) of the median, underweight as WAZ of <−2 SDs, and wasting as WLZ < −2 SDs; further, overweight was defined as WLZ > 2 SDs. There were no unified standards to assess normal mineral contents in hair of infants and young children. So, we mainly compared relative levels between groups.

Four indicators recommended by WHO were used to evaluate the complementary feeding status of IYC [24]: (1) introduction of solid, semi-solid, or soft foods (ISSF): proportion of infants 6–8 months of age who receive solid, semi-solid, or soft food; (2) minimum dietary diversity (MDD): proportion of children 6–23 months of age who receive foods from 4 or more food groups; (3) minimum meal frequency (MMF): proportion of breastfed and non-breastfed children 6–23 months of age who receive solid, semi-solid, or soft foods (but also including milk feeds for non-breastfed children) the minimum number of times or more; and (4) minimum acceptable diet (MAD): proportion of children 6–23 months of age who have at least the minimum dietary diversity and the minimum meal frequency (apart from breast milk).

### 2.5. Data Handling and Analysis

EpiData3.1 was used to establish the database and conduct double-entry verification. All data of individuals with missing key information were excluded from statistical analysis. Taking mean ± 5 SDs as the boundary point, individuals whose data indicators were not within this range or missing were not included in the analysis of this indicator. Statistical analysis was performed using SPSS (version 19.0; IBM Institute). Data were reported as the mean ± SDs for continuous variables and as number (frequencies) for categorical variables. If continuous variables were normally distributed, analysis of variance (ANOVA) was performed to analyze the differences between groups; if not, Mann–Whitney U tests were performed. Categorical variables were analyzed by chi-square test. Spearman correlation coefficient (r_s_) was used to indicate the correlation between the two variables. Differences were considered statistically significant at *p* < 0.05.

## 3. Results

### 3.1. Demographic Characteristics

The basic demographic characteristics of the IYC, their parents, and caregivers are shown in Table 1. A total of 1522 IYC were included in this study, including 795 boys (52.1%) and 731 girls (47.9%). The average age was 15.61 months. The average ages of mothers and fathers were 28.87 and 31.26 years, respectively. About 60% of the parents were Han, and the others were ethnic minorities. Most parents completed junior middle school; 47.4% of mothers were homemakers, while only 2.6% of fathers were unemployed; 75.4% of IYC were taken care of by their parents, and 26.4% by their grandparents or other relatives. Most caregivers completed junior middle school, and nearly 60% of caregivers were not employed.

### 3.2. Grouping Results

A two-step cluster analysis was carried out to automatically classify the 1515 IYC into three clusters according to the z-scores value. Table 2 shows the characteristics of the clusters. The average values of LAZ, WAZ, and WLZ of IYC aged 6–23 months in rural areas of China were −0.23, −0.09, and 0.05, respectively, with 7.1%, 3.0%, and 3.7% prevalence of stunting, wasting, and overweight, respectively. There were significant differences in the average values of LAZ, WAZ, and WLZ between any two clusters (*p* < 0.01). The nutritional status of the IYC in Cluster 2 was the best, and there was no malnutrition prevalence. Cluster 1 had a relatively high prevalence of stunting, underweight, and wasting, and the proportion of undernutrition was higher than that in Clusters 2 and 3 (*p* < 0.01). The WLZ of Cluster 3 was significantly higher than that of the other two clusters (*p* < 0.01), and the overweight was up to 12.2%.

**Figure 1:**
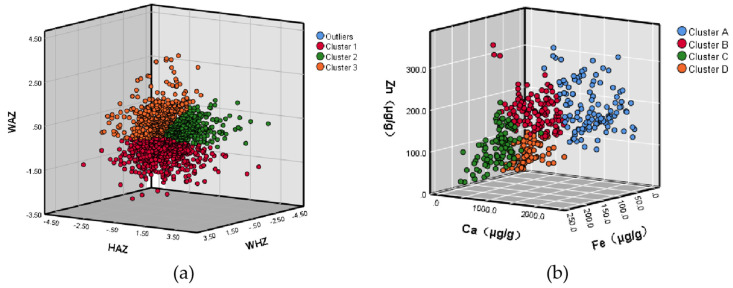
Grouping Results. Clusters categorized according to z-scores (**a**), or to Ca, Fe, and Zn levels in the hair (**b**).

The Ca, Fe, and Zn contents in the hair of 428 IYC were determined. The median content of Ca, Fe, and Zn in the hair of 6–23-month-old IYC in rural areas in 2018 were 550.10 µg/g, 62.94 µg/g, and 132.86 µg/g, respectively. A two-step cluster analysis was performed based on the measurement results. The cluster distribution and Ca, Fe, and Zn contents expressed as median level and interquartile range are shown in Table 3. The median contents of Ca, Fe, and Zn in hair of Cluster B were in line with the overall level. The median Ca content in hair of Cluster A was significantly higher than that of the other three clusters, while the Fe content was significantly lower (*p* < 0.01). The median content of Ca in hair of Cluster C was the lowest, while that of Fe was the highest, and the content of Zn was lower than that in clusters A and B (*p* < 0.01). The median content of Fe in hair in Cluster D was consistent with that in Cluster B and the overall level; however, the content of Zn was the lowest among the four clusters (*p* < 0.01). In addition, it is worth noting that the Ca content in IYC hair was positively correlated with the Zn content (r_s_ = 0.517, *p* < 0.01) but negatively correlated with the Fe content (r_s_ = −0.742, *p* < 0.01), as presented in Table A1. Cluster distributions are shown in Figure 1.

### 3.3. Complementary Feeding Status

The complementary feeding statuses of IYC between different clusters are shown in Table 4 and Table 5. IYC with different z-scores had different proportions and structures of complementary foods. As listed in Table 4, IYC in good nutrition status (Cluster 2) consumed more dairy products than undernourished IYC (Cluster 1). The type of complementary food added to infants with a higher WLZ (Cluster 3) was relatively single. IYC in Cluster 3 was fed fewer grains, roots, and tubers than IYC in Clusters 1 and 2 (*p* < 0.01). The prevalence of ISSF, MDD, MMF, and MAD was lowest in IYC with a higher WLZ. Overall, the prevalence of meeting the requirements for the introduction of solid, semi-solid, or soft foods (ISSF), minimum dietary diversity (MDD), minimum meal frequency (MMF), and minimum acceptable diet (MAD) were 85.2%, 68.9%, 77.9%, and 46.4%, respectively.

There was no significant difference in CF status between IYC with different contents of Ca, Fe, and Zn in hair.

## 4. Discussion

This study investigated the nutritional status and complementary feeding of IYC aged 6–23 months in rural areas of China in 2018. According to the epidemic threshold of wasting, overweight, and stunting among children under 5, as determined by the WHO and UNICEF nutrition monitoring technical advisory group [25], the prevalence of malnutrition and overweight among 6–23-month-old IYC in rural areas in China was at a “low” level in 2018.

The ISSF (6–8 months), MDD, MMF, and MAD of the IYC were compared in different nutritional statuses. It was found that the proportion of dairy products added to complementary foods for the well-developed IYC (Clusters 2) was higher than other clusters. Milk is rich in minerals, with an average of 120 mg calcium per 100 mL of milk, and has a high absorption rate. It is a good source of various minerals and high-quality proteins. In Europe and North America, approximately 75% of dietary calcium comes from dairy products. A diet including dairy products is considered the best choice for calcium concentration to prevent adverse health effects associated with negative calcium balance [26]. The prevalence of MDD and MAD of IYC in Cluster 3 was lower than that of the other clusters, and the proportions of added grains, roots, and tubers in the complementary foods were significantly low. When adding complementary foods, grains are the first choice for IYC because they are easy to digest and can hardly cause allergic reactions [27]. Several studies have shown that increasing whole grain intake can reduce the risk of obesity and diabetes in adults [28,29,30]. The nutritional characteristics of different foods differ. Only by intake of a diverse diet can comprehensive nutrition be ensured. *The Chinese Nutrition Guidelines of Complementary Feeding for Infants and Toddlers (WS/T 678-2020)* suggests that the groups of complementary foods for IYC should gradually change from single to diverse. IYC are fed one additional new food each time, from less to more, and, finally, fed four or more of the seven common foods every day [27]. Our findings on the importance of complementary feeding were particularly consistent with results from studies conducted in other countries. Petrikova, I. [31] found that consuming more vitamin-A-rich fruits and vegetables significantly reduced the risk of underweight and wasting in 6- to 23-month-old children of India, and eating any meat would lower the likelihood of being stunted or underweight. Crabben, S. et al. [32] deemed that the increase in the percentage of stunted growth in Bush Negro infants in the interior of Surinam was better explained by a shortage of well-balanced complementary feeding rather than by an absolute shortage of energy. In addition, Nai, H.M.E. et al. [33] found that poor dietary diversity of complementary foods was a risk factor of stunting among children aged 6–23 months. There are also different findings from studies in other nationals. For example, Tang, M. et al. [34] found that LAZ significantly decreased in the dairy complementary food group compared with the meat group. This may be explained by different amounts of intakes. Moreover, further investigation is clearly warranted.

In addition, this survey showed that the mineral content in infants’ hair aged 6–23 months varies greatly among individuals. The proportion and structure of complementary foods among the IYC with different hair micronutrients showed no significant difference. Perhaps more rigorous design, larger sample sizes, and more sensitive indicators are needed to see significant differences. Wang, J. et al. [35] found that diverse diet is a protective factor against micronutrient deficiency in children over 12 months old.

MNDs are widespread among infants and preschool children in low- to middle-income countries. Iron deficiency is the most prevalent MND worldwide. Iron deficiency can lead to microcytic anemia and impair immune and endocrine functions [17]. Both Ca and Zn are important elements that promote growth and development. Zn deficiency causes calcium deposition in the bone, resulting in bone growth retardation, which seriously affects IYC growth and development. Changes in zinc homeostasis also affect immune function [36,37]. Zinc supplements have been widely used and approved for the clinical treatment of various diseases. For example, zinc is very effective in treating infantile diarrhea [36]. A clinical study on plasma trace element levels in adults with dyslipidemia in Serbia found a significant inverse relationship between plasma zinc concentration and BMI and obesity [38]. Breast milk alone cannot meet the needs of children aged over 6 months [39]. Therefore, complementary foods rich in Fe and Zn must be added promptly. *The Dietary Guidelines for Chinese Residents (2016)* recommend that infants start with Fe-rich muddy food and gradually achieve food diversity [40]. For infants lacking Ca, Fe, Zn, and other micronutrients, appropriate amounts of milk, red meat, and animal organ meat should be fed. Plant-based diets usually cannot provide sufficient critical micronutrients to meet the recommended nutrient intake for 6–23-month-old IYC [41]. Varieties of vitamins, such as vitamin C, which can promote the absorption of Fe, and vitamin D3, which can promote the absorption of Ca and Zn, should be supplemented actively.

Many low- and middle-income countries have implemented strategies to prevent MNDs in children under five [42] using multiple micronutrient powders to fortify foods eaten by IYC aged 6–23 months at home [41], such as multiple-micronutrient (MMN) supplementation for women during pregnancy [43,44,45], small-quantity lipid-based nutrient supplementation (SQ-LNS) [46], and targeted large-scale or point-of-use fortified micronutrient powders (MNPs) [47,48]. China’s ongoing YYB policy has achieved excellent results [49,50]. Since 2000, the incidence of malnutrition in China has decreased steadily. The Nutrition Improvement Project on Children in Poor Areas of China (NIPCPAC) provides nutritional and technical support to low-income families. Our study provides scientific data for the implementation of nutrition intervention policies, and specific and implementable references for strengthening primary health care for children. Thus, more effective measures will be put forward to improve the health condition of infants and young children in rural areas of China and narrow the urban–rural gaps.

Our study has several strengths. The latest complementary feeding (CF) indicators were used to assess the prevalence of qualified feeding in the infants and young children (IYC) in rural China, and a two-step cluster analysis was applied to cluster the IYC. Additionally, we not only evaluated and compared the differences in complementary feeding structure of IYC with different nutritional status, but also analyzed the differences in their intake of specific food species. Besides, we were concerned about micronutrient deficiencies (MNDs) as well as protein-energy malnutrition.

Our study also has its limitations. Most importantly, hair is chosen as the matrix because of its advantages: sampling is not painful or invasive; however, there is no global or national survey on micronutrient deficiencies (MNDs) in children under 2 years old, let alone unified reference standards for the normal range [51]. In addition, we simply collected the frequency of seven groups of complementary foods, while the amount of food consumed was not included. Finally, we did not delve into the underlying causes of infants’ dietary habits; thus, future studies could focus more on this aspect.

## 5. Conclusions

The prevalence of anthropometric deficits in the 6–23-month-old IYC in rural areas of China was low in 2018, but MNDs remained serious. A good dietary pattern was proved beneficial to maintain balanced nutrition and health. The prevalence of meeting the requirements for MAD was surprisingly low, especially in overweight children and children with low levels of Ca and Zn in their hair. To further improve the nutritional status of IYC and prevent malnutrition, more attention should be paid to complementary feeding and micronutrient-related malnutrition.

## Figures and Tables

**Table 1:** Basic demographic characteristics.

Characteristics	Mean or Frequency (SD or Rate)
**IYC**	
Age, month	15.6 (5.4)
Male, *n* (%)	795 (52.1)
Birth Length, cm	50.2 (1.7)
Birth Weight, g	3290.4 (501.2)
**Mother**	
Age, year	28.9 (5.2)
Han, *n* (%)	907 (59.4)
Education	
Primary School or Below, *n* (%)	171 (11.2)
Middle School, *n* (%)	992 (65.1)
High School or Above, *n* (%)	361 (20.1)
Domestically Unemployed, *n* (%)	722 (47.4)
**Father**	
Age, year	31.3 (5.5)
Han, *n* (%)	948 (62.1)
Education	
Primary School or Below, *n* (%)	133 (8.7)
Middle School, *n* (%)	1009 (66.2)
High School or Above, *n* (%)	382 (25.1)
Domestically Unemployed, *n* (%)	40 (2.6)
**Caregiver**	
Parents as main caregivers, *n* (%)	1151 (75.4)
Education	
Primary School or Below, *n* (%)	422 (28.0)
Middle School, *n* (%)	847 (55.6)
High School or Above, *n* (%)	167 (16.4)
Domestically Unemployed, *n* (%)	898 (59.0)

IYC: infants and young children.

**Table 2:** Clusters grouped by z-scores.

Clusters	Distribution *n*(%)	Z-Score x ± SD			The Prevalence of Malnutrition *n* (%)			
		LAZ	WAZ	WLZ	Stunting	Underweight	Wasting	Overweight
1	680 (44.9)	−1.06 ± 0.89	−1.00 ± 0.59	−0.63 ± 0.81	98 (14.4)	44 (6.5)	44 (6.5)	0 (0.0)
2	377 (24.9)	0.99 ± 0.82	0.33 ± 0.46	−0.17 ± 0.56	0 (0.0)	0 (0.0)	2 (0.5)	0 (0.0)
3	458 (30.2)	−0.01 ± 1.01	0.92 ± 0.80	1.25 ± 0.68	13 (2.8)	0 (0.0)	0 (0.0)	56 (12.2)
Total	1515 (100.0)	−0.23 ± 1.23	−0.09 ± 1.06	0.05 ± 1.08	111 (7.3)	44 (2.9)	46 (3.0)	56 (3.7)

LAZ: length-for-age z-score. WAZ: weight-for-age z-score. WLZ: weight-for-length z-score. Stunting, underweight and wasting was defined as LAZ, WAZ and WLZ < −2 SDs. Overweight was de-fined as WLZ > 2 SDs.

**Table 3:** Clusters grouped by micronutrient contents in hair.

Clusters	Distribution *n*(%)	Micronutrient in Hair M(Q)		
		Ca (µg/g)	Fe (µg/g)	Zn (µg/g)
A	114 (26.6)	1462.35 (598.82)	24.47 (20.50)	173.47 (78.92)
B	125 (29.2)	607.50 (385.88)	61.50 (35.79)	164.48 (48.88)
C	103 (24.1)	248.94 (166.60)	115.26 (35.60)	92.33 (49.68)
D	86 (20.1)	445.70 (219.79)	66.58 (32.72)	59.78 (43.65)
Total	428 (100.0)	550.10 (748.38)	62.94 (57.55)	132.86 (93.01)

**Table 4:** The CF status in IYC with different z-scores.

	Clusters			Total	*p*
	1 (Undernutrition)	2 (Fine)	3 (Overweight)		
**Groups of CF**					
Grains, roots, and tubers	651 (96.9)	355 (95.9)	410 (92.6)	1416 (95.4)	<0.01
Vitamin-A-rich fruits and vegetables	429 (63.9)	248 (67.0)	262 (59.1)	939 (63.3)	0.06
Other fruits and vegetables	448 (66.8)	248 (67.0)	281 (63.4)	977 (65.8)	0.44
Fresh foods	392 (58.3)	197 (53.2)	229 (51.7)	818 (55.1)	0.06
Eggs	322 (47.9)	185 (50.0)	216 (48.8)	723 (48.7)	0.81
Dairy products	397 (58.4)	257 (68.2)	261 (57.0)	915 (60.4)	<0.01
Legumes and nuts	288 (42.9)	145 (39.2)	176 (39.7)	609 (41.0)	0.42
**Structure of CF**					
ISSF (6–8 months)	56 (86.2)	39 (86.7)	60 (83.3)	155 (85.2)	0.85
MDD	484 (71.2)	274 (72.9)	285 (62.2)	1043 (68.9)	<0.01
MMF	469 (78.3)	274 (81.5)	297 (74.3)	1040 (77.9)	0.05
MAD	251 (49.5)	157 (49.7)	145 (39.4)	553 (46.4)	<0.01

CF: complementary feeding. ISSF: introduction of solid, semi-solid, or soft foods. MDD: minimum dietary diversity. MMF: minimum meal frequency. MAD: minimum acceptable diet.

**Table 5:** The CF status in IYC with different micronutrient contents in hair.

	Clusters				Total	*p*
	A (High Ca and Low Fe)	B (Fine)	C (Low Ca, Low Zn, and High Fe)	D (Low Ca and Low Zn)		
**Groups of CF**						
Grains, roots, and tubers	101 (93.5)	115 (95.0)	97 (98.0)	78 (94.0)	391 (95.1)	0.43
Vitamin-A-rich fruits and vegetables	58 (53.7)	65 (53.7)	58 (59.2)	52 (62.7)	233 (56.8)	0.52
Other fruits and vegetables	67 (62.0)	92 (76.0)	67 (68.4)	54 (65.1)	280 (68.3)	0.13
Fresh foods	64 (59.3)	63 (52.1)	51 (51.5)	41 (49.4)	219 (53.3)	0.52
Eggs	52 (48.1)	61 (50.4)	44 (44.4)	38 (45.8)	195 (47.4)	0.84
Dairy products	73 (64.0)	82 (65.6)	63 (61.2)	51 (59.3)	269 (62.9)	0.79
Legumes and nuts	43 (39.8)	55 (45.5)	48 (48.5)	44 (53.0)	190 (46.2)	0.31
**Structure of CF**						
ISSF (6–8 months)	14 (70.0)	10 (83.3)	4 (66.7)	8 (72.7)	36 (73.5)	0.83
MDD	72 (63.2)	87 (69.6)	66 (64.1)	60 (69.8)	285 (66.6)	0.62
MMF	75 (75.8)	98 (84.5)	74 (77.9)	53 (73.6)	300 (78.5)	0.27
MAD	40 (46.0)	56 (52.8)	28 (37.8)	30 (44.8)	154 (46.1)	0.26

CF: complementary feeding. ISSF: introduction of solid, semi-solid, or soft foods. MDD: minimum dietary diversity. MMF: minimum meal frequency. MAD: minimum acceptable diet.

## Data Availability

Not applicable.

## Appendix A

**Table A1:** Correlations between micronutrient contents in hair.

	Spearman Correlation	*p*
Ca vs. Zn	0.517	<0.01
Ca vs. Fe	−0.742	<0.01
Zn vs. Fe	−0.461	<0.01
